# Two Major Amputations

**Published:** 1894-09

**Authors:** Hunter P. Cooper

**Affiliations:** Attending Surgeon, Grady Hospital, Atlanta, Ga.


ATLANTA

### MEDICAL AND SURGICAL JOURNAL.

VOL. XI. SEPTEMBER, 1894. No. 7.

EDITED BY

LUTHER B. GRANDY, M.D., AND MILLER B. HUTCHINS, M.D.,

WITH THE CO-OPERATION OF

H. V. M. MILLER, M.D., LL.D., VIRGIL O. HARDON, M.D.,
FLOYD W. McRAE, M.D., ARTHUR G. HOBBS, M.D.,
AND H. R. SLACK, M.D., PH.M.

### *ORIGINAL COMMUNICATIONS.*

### TWO MAJOR AMPUTATIONS.

1. AMPUTATION AT THE HIP JOINT BY WYETH'S BLOODLESS METHOD. 2. AMPUTATION AT THE SHOULDER JOINT.

BY HUNTER P. COOPER, M.D.,
Attending Surgeon, Grady Hospital, Atlanta, Ga.

It is not often that cases of amputation at the hip joint are reported, and for this reason I think this case will possess some interest, particularly as it was done by Wyeth's bloodless method.

The patient, J. W., age sixteen, negro, was admitted to my service in the Grady Hospital on June 26, 1894, with the following history:

Three years ago he received a very severe contusion of the knee, which caused the joint to be painful for a month or more; then he recovered, but the knee joint was permanently stiffened. Two months ago the knee began to pain him again, swelled up rapidly, and abscesses formed about the knee and thigh. Pieces of bone had come out of these abscess cavities previous to his admission.

The patient was in a deplorable condition, very much emaciated, tongue foul and coated, and bowels constipated. The left knee was enormously distended with pus, and there were two or three open sinuses at various points about the knee joint which were discharging rather freely. Flexion of the knee joint was very limited and exceedingly painful. There was evidently total destruction of the internal lateral ligament, as the leg could be easily abducted over a considerable angle.

There was also an open sinus midway between the knee and greater trochanter, which led straight down to the femur, and at this point a probe detected dead bone. The whole femur was enlarged to probably double its natural size from the knee to the greater trochanter, and very tender to pressure. The lower group of inguinal glands was very much enlarged. The diagnosis was suppurative arthritis of the knee joint, with osteitis of the whole shaft of the femur, and it was considered highly probable that this inflammation was of a tubercular character.

The boy's general health was so bad that a radical operation at that time was out of the question; so that I merely contented myself by etherizing him and making free openings into the joint, putting in through drainage and washing it out thoroughly. At the same time I excised the group of enlarged glands in the groin, and sewed the wound up. The knee was dressed with a thick antiseptic dressing and put on a posterior splint. Under this treatment the fever which he had been having at once disappeared, and under generous diet and stimulants he rapidly began to build up in strength and flesh.

It was evident that nothing of a permanent nature could be done for him except an amputation at the hip joint, as the condition of the femur precluded the idea of a resection of the knee. On July 11th I considered that he was ready to have the amputation done, and it was accordingly done with the kind assistance of Drs. Elkin and Nicolson and the resident staff of the Grady Hospital.

The field of operation having been rendered aseptic, the mattress needles were introduced according to Wyeth's method. The needles used were the largest I could buy, being nearly three-sixteenths of an inch in diameter and a little over twelve inches long. The first needle was entered an inch below and a little to the inner side of the anterior superior spine of the ilium, and carried through the tissues so that its point emerged just back of the greater trochanter. The other needle was entered at a point about an inch below the level of the crotch and internal to the saphenous opening and passed through the adductor muscles, so that its point came out about an inch in front of the tuberosity of the ischium. Above these needles the rubber tourniquet was tightly wound twice around the limb and secured. I then made a circular incision about four inches below the rubber tourniquet, and dissected up a cuff consisting of all the tissues down to the deep fascia. This was reflected, and a circular incision made through the muscles down to the bones at the level of the lesser trochanter. In order to get a little better leverage for disarticulating the head of the bone, I then stripped the muscles from the femur below the point at which the circular incision had been made, and sawed the bone at a point about three or four inches below this point. This furnished an admirable handle for flexing and rotating the bone in order to dislodge the head from the acetabulum. The main vessels were next secured, the tourniquet removed, and such vessels as were bleeding quickly tied. All muscular attachments to the upper end of the femur were then divided, and the end of the femur drawn upwards and inwards strongly in the direction of the patient's navel.

The knife was then introduced posterior to the head of the bone and the cotyloid ligament divided. This allowed the bone to be easily disarticulated. The wound was then stitched up, two drainage tubes inserted and a heavy antiseptic dressing applied. So nearly as we could estimate, the patient did not lose more than an ounce or two of blood. He rallied very nicely from the operation, and for the next seven days his temperature did not get as high as 99°, except on two occasions. The dressings were changed for the first time on the seventh day. Union took place by first intention throughout the wound, except, of course, at the points where the drainage-tubes were introduced, and these points rapidly healed up after removal of the drainage-tubes without discharging a particle of pus.

During his convalescence he had a sharp attack of acute dysentery, and for that reason I kept him in the hospital longer than I otherwise would have done. He was discharged August 4th well and strong.

The next case is one of amputation at the shoulder joint for sarcoma of the humerus. The patient, T. C., age eighteen, white, was admitted to the Grady Hospital June 11, 1894. He said that one year ago he felt a pain in his left arm just above the elbow. The arm at this point began to swell, the pain increasing in severity, and this went on until the whole arm from just above the elbow to within four inches of the shoulder was involved in a large and hard swelling which was exceedingly painful.

He came to the hospital two weeks before admission, and was advised to have an amputation done, but would not consent to the operation. On June 11th, while trying to get out of a chair, he put slight pressure on his left arm and felt something give away in the growth. He came direct to the hospital in great pain. He was supporting the left arm in his right hand in order to steady it, as the slightest motion caused excessive pain. The whole arm and the growth were enveloped in a large, soft pillow, which was bandaged in place, as it was discovered that there was a fracture of the humerus within the growth. When I first saw him the following day he had a temperature of 100½°, and the left arm was seen to be the seat of a large sarcoma, involving all the tissues from the bone to the skin.

As it was not convenient for me to operate on him that day, I postponed the operation until the following day. On June 13th his temperature just previous to the operation was nearly 104°, produced apparently by some infection at the seat of fracture.

The patient was etherized and the arm amputated at the shoulder joint by Larrey's method. The circulation was completely controlled by compressing the subclavian with a padded compress over the first rib. This was done during the operation by Dr. Campbell, the house surgeon. Dr. Nicolson followed the flap down as I removed the arm, to give additional security against hemorrhage.

The wound was first dressed on fifth day, and it was found that union by first intention had occurred everywhere, except where three small stitch abscesses had formed. These stitches were removed and this caused a little separation of the skin, and at this point healing took place by granulation.

At the end of two weeks he was up attending to duties around the ward. He was kept in the hospital for some time on account of the slowness with which granulation took place at the points where the stitches had been removed. He was discharged well August 3d.

